# Probing the binding site of novel selective positive allosteric modulators at the M_1_ muscarinic acetylcholine receptor

**DOI:** 10.1016/j.bcp.2018.05.009

**Published:** 2018-08

**Authors:** Elham Khajehali, Celine Valant, Manuela Jörg, Andrew B. Tobin, P. Jeffrey Conn, Craig W. Lindsley, Patrick M. Sexton, Peter J. Scammells, Arthur Christopoulos

**Affiliations:** a Drug Discovery Biology, Monash Institute of Pharmaceutical Sciences, Monash University, 381 Royal Parade, Parkville, VIC 3052, Australia; b Medicinal Chemistry, Monash Institute of Pharmaceutical Sciences, Monash University, 381 Royal Parade, Parkville, VIC 3052, Australia; c Centre for Translational Pharmacology, Institute of Molecular, Cell and Systems Biology, College of Medical, Veterinary and Life Sciences, University of Glasgow, Glasgow G12 8QQ, United Kingdom; d Department of Chemistry, Department of Pharmacology, Vanderbilt Center for Neuroscience Drug Discovery, Nashville, TN 37232, United States

**Keywords:** ACh, acetylcholine, mAChR, muscarinic acetylcholine receptor, GPCR, G protein-coupled receptor, AD, Alzheimer’s disease, PAM, positive allosteric modulator, NAM, negative allosteric modulator, TM, transmembrane domain, ECL, extracellular loop, CHO, Chinese hamster ovary, WT, wild type, DMEM, Dulbecco modified eagle medium, FBS, fetal bovine serum, BSA, bovine serum albumin, PBS, phosphate-buffered saline, HBSS, Hanks’ balanced salt solution, IP_1_, inositol monophosphate, NMS, *N*-methylscopolamine, PBZ, phenoxybenzamine, BQCA, benzylquinolone carboxylic acid, 1-(4-methoxybenzyl)-4-oxo-1,4-dihydroquinoline-3-carboxylic acid, PF-06767832, *N*-((3*R*,4*S*)-3-hydroxytetrahydro-2*H*-pyran-4-yl)-5-methyl-4-(4-(thiazol-4-yl)benzyl)pyridine-2-carboxamide, VU6004256 (4, 6-difluoro-*N*-(1*S*,2*S*)-2-hydroxycyclohexyl-1-((6-(1-methyl-1*H*-pyrazol-4-yl)pyridine-3-yl)methyl)-1*H*-indole-3-carboxamide, MIPS1780, 3-(2-hydroxycyclohexyl)-6-(2-((4-(1-methyl-1*H*-pyrazol-4-yl)- benzyl)oxy)phenyl)pyrimidin-4(3*H*)-one, BQZ-12, 3-((1*S*,2*S*)-2-hydroxycyclohexyl)-6-((6-(1-methyl-1*H*-pyrazol-4-yl)pyridin-3-yl)methyl)benzo [*h*]-quinazolin-4(3*H*)-one, ANOVA, analysis of variance, Allosteric modulation, Muscarinic acetylcholine receptor, BQCA, Mutagenesis, Drug discovery

## Abstract

Subtype-selective allosteric modulation of the M_1_ muscarinic acetylcholine (ACh) receptor (M_1_ mAChR) is an attractive approach for the treatment of numerous disorders, including cognitive deficits. The discovery of benzyl quinolone carboxylic acid, BQCA, a selective M_1_ mAChR positive allosteric modulator (PAM), spurred the subsequent development of newer generation M_1_ PAMs representing diverse chemical scaffolds, different pharmacodynamic properties and, in some instances, improved pharmacokinetics. Key exemplar molecules from such efforts include PF-06767832 (*N*-((3*R*,4*S*)-3-hydroxytetrahydro-2*H*-pyran-4-yl)-5-methyl-4-(4-(thiazol-4-yl)benzyl)pyridine-2-carboxamide), VU6004256 (4,6-difluoro-*N*-(1*S*,2*S*)-2-hydroxycyclohexyl-1-((6-(1-methyl-1*H*-pyrazol-4-yl)pyridine-3-yl)methyl)-1*H*-indole-3-carboxamide) and MIPS1780 (3-(2-hydroxycyclohexyl)-6-(2-((4-(1-methyl-1*H*-pyrazol-4-yl)-benzyl)oxy)phenyl)pyrimidin-4(3*H*)-one). Given these diverse scaffolds and pharmacodynamics, the current study combined pharmacological analysis and site-directed mutagenesis to explore the potential binding site and function of newer M_1_ mAChR PAMs relative to BQCA. Interestingly, the mechanism of action of the novel PAMs was consistent with a common model of allostery, as previously described for BQCA. Key residues involved in the activity of BQCA, including Y179 in the second extracellular loop (ECL) and W400^7.35^ in transmembrane domain (TM) 7, were critical for the activity of all PAMs tested. Overall, our data indicate that structurally distinct PAMs share a similar binding site with BQCA, specifically, an extracellular allosteric site defined by residues in TM2, TM7 and ECL2. These findings provide valuable insights into the structural basis underlying modulator binding, cooperativity and signaling at the M_1_ mAChR, which is essential for the rational design of PAMs with tailored pharmacological properties.

## Graphical abstract

**Figure:**
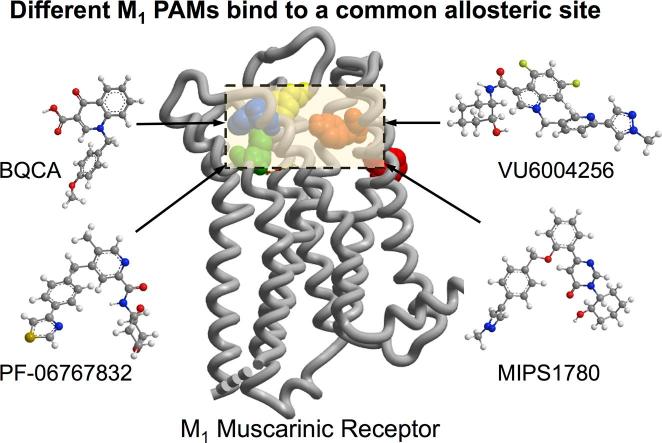

## 1 Introduction

Muscarinic acetylcholine receptors (mAChRs) are members of the Class A G protein-coupled receptor (GPCR) family [1] involved in central and peripheral biology [2]. Five mAChR subtypes (M_1_-M_5_), have been identified; M_1_, M_3_ and M_5_ mAChRs preferentially couple to G_q/11_ proteins; M_2_ and M_4_ mAChRs preferentially couple to G_i/o_ proteins [3]. Of note, the M_1_ mAChRs are highly expressed in forebrain regions, including the cerebral cortex, hippocampus and striatum [4]. Transgenic M_1_ mAChR studies implicated roles for this receptor in neuronal excitability, locomotor activity and learning and memory [Reviewed in [5]]. Therefore, selective activation of the M_1_ mAChR has emerged as an approach for the treatment of cognitive deficits associated with disorders such as Alzheimer’s disease (AD) and schizophrenia [6], [7]. This is of particular relevance due to limitations associated with current cognition-enhancing agents [8]. For instance, loss of cholinergic neurons is a hallmark of AD [9], [10] and inhibitors of acetylcholinesterase remain the primary treatment for disease symptoms [11] yet are associated with substantial adverse effects [12]. Unfortunately, drug discovery efforts aimed at developing directly acting M_1_ mAChR agonists have been unsuccessful. The best clinical example is the M_1_/M_4_-preferring agonist xanomeline, which proved beneficial in improving cognitive function and psychotic symptoms in AD and schizophrenia [13], but was not further developed due to off-target gastrointestinal adverse effects from interaction with other mAChRs [14].

An alternative approach to developing subtype-selective drugs is through targeting topographically distinct allosteric sites [15], [16]. In this regard, the discovery of benzyl quinolone carboxylic acid (BQCA; 1-(4-methoxybenzyl)-4-oxo-1,4-dihydroquinoline-3-carboxylic acid), a selective M_1_ positive allosteric modulator (PAM) was a major development for the field [17], leading to additional M_1_ mAChR PAMs. However, BQCA has a low affinity for the M_1_ mAChR [18], and was associated with liabilities that precluded further development, such as poor brain penetration and solubility, and high plasma protein binding [19]. The work with BQCA also highlighted complexities associated with design of allosteric modulators as potential therapeutics. In general, such challenges are two-fold. The first relates to understanding of the molecular properties associated with allosteric drugs, including affinity, cooperativity with the orthosteric agonist (positive or negative), and whether the allosteric ligand possesses intrinsic signaling efficacy; the interplay between these properties is only starting to be appreciated, and varies depending on the target and disease [20]. The second major challenge for discovery programs is optimizing physicochemical/pharmacokinetic properties of candidate molecules to ensure appropriate target coverage when/where required, while minimizing off-target activity. A key development in this regard is the identification of new allosteric scaffolds with which to target GPCRs. For instance, Pfizer recently disclosed a novel M_1_ mAChR ‘PAM-agonist’, PF-06767832, with promising physiochemical and pharmacokinetic properties. However, PF-06767832 exhibited seizure liability, and cardiovascular and gastrointestinal side-effects, indicating that M_1_ mAChR over-activation may directly contribute to adverse events [21]. A more recent study using PAMs with reduced intrinsic allosteric agonist activity also noted adverse effects [22]. This may reflect deficiencies in our understanding of the ‘optimal’ degree of positive cooperativity required for specific disease intervention [18], [23]. An alternative selective M_1_ PAM-agonist, VU6004256, reversed cognitive deficits in a mouse model, indicative of preclinical efficacy [24]. The chemotype and molecular pharmacology of this M_1_ PAM-agonist is similar to PF-06764427, yet they display different *in vivo* activities [25]. Our recent structure-activity studies also led to discovery of another series of M_1_ mAChR PAMs [26], with a key exemplar being MIPS1780 (compound **29** in [26]).

**Fig. 1:**
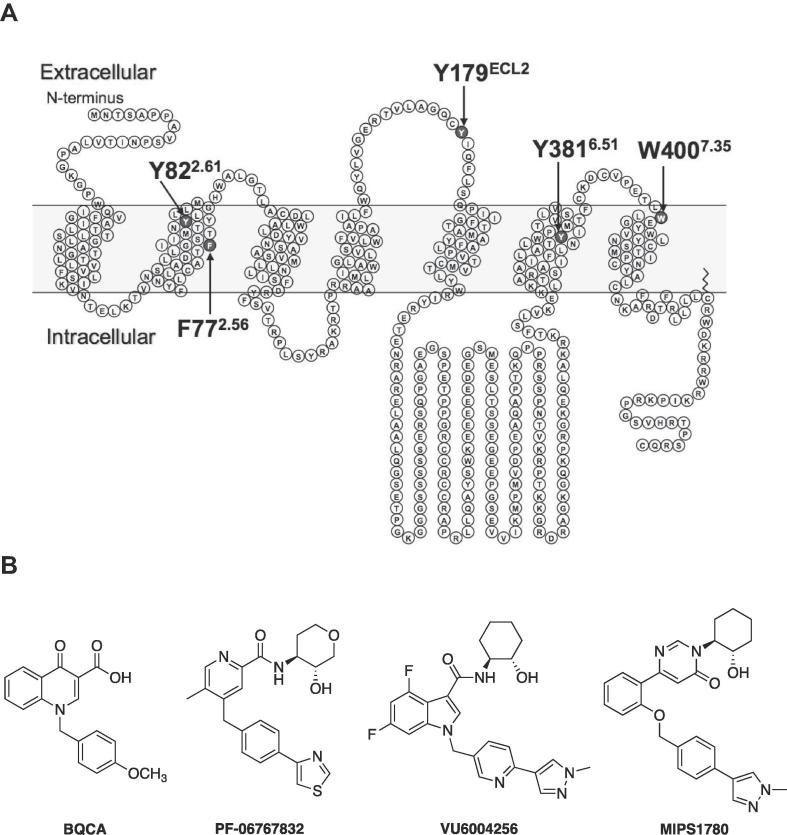
Mutations and ligands investigated in the current study. (A) A snake diagram of the hM_1_ mAChR highlighting the mutated residues and (B) chemical structures of the M_1_ positive allosteric modulators used in the current study.

However, despite the increasing availability of a range of novel M_1_ mAChR PAM scaffolds with different molecular and functional properties, the extent to which their differential activities are driven from interaction with a common allosteric binding pocket, or from alternative regions is not known. Previously, we utilized mutagenesis to reveal that BQCA binds to a “common” allosteric mAChR binding site, located in an extracellular vestibule defined by residues predominantly in TM2, TM7 and ECL2 [27]. However, there is also pharmacological evidence that the mAChRs possess at least a second allosteric site [28], [29], [30]. Thus, the aim of the current study was to apply a combination of analytical pharmacology and site-directed mutagenesis (Fig. 1A) to explore the potential binding site and function of novel selective M_1_ mAChR PAMs with diverse scaffolds, PF-06767832, VU6004256 and MIPS1780, in comparison to the first-generation BQCA (Fig. 1B). Our results provide evidence that these ligands act solely as modulators of ACh affinity, not efficacy, and may bind to a similar binding pocket at the M_1_ mAChR and exert their effects with subtle differences, but largely consistent, with those of BQCA. This provides valuable insight into the structural basis underlying allosteric ligand binding and function at the M_1_ mAChR.

## 2 Materials and methods

### 2.1 Materials

Dulbecco’s modified Eagle’s medium (DMEM) and fetal bovine serum (FBS) were purchased from Invitrogen (Carlsbad, CA) and ThermoTrace (Melbourne, Australia), respectively. Hygromycin B was purchased from Roche (Mannheim, Germany). IP-One assay kit and reagents were purchased from Cisbio (Codolet, France). [^3^H]*N*-methylscopolamine ([^3^H]NMS); specific activity, 75 Ci/mmol) and Ultima gold and MicroScint scintillation liquid were purchased from PerkinElmer Life Sciences (Boston, MA). BQCA and MIPS1780 were synthesized in-house at the Monash Institute of Pharmaceutical Sciences as described previously [26], [31]. VU6004256 was synthesized in-house at Vanderbilt University as described previously [25]. All other chemicals were purchased from Sigma Aldrich (St. Louis, MO), or as otherwise stated below. FlpIn Chinese Hamster Ovary (CHO) cells stably expressing the wild type (WT) or mutant c-myc hM_1_ mAChRs (passage numbers 8–25) were generated as described previously [32], and maintained in DMEM supplemented with 5% FBS, 16 mM HEPES and 600 μg/ml hygromycin B at 37 °C in humidified atmosphere containing 5% CO_2_.

### 2.2 Whole cell radioligand binding assays

Saturation binding assays were performed to estimate the expression levels and equilibrium dissociation constant of the radioligand (K_D_). FlpIn CHO cells stably expressing the WT or mutant c-myc hM_1_ mAChRs were plated at the density of 25,000 per well of 96-well white clear bottom Isoplates (PerkinElmer Life Sciences, Boston, MA), and grown overnight at 37 °C. The following day, cells were washed twice with Phosphate Buffer Saline (PBS), and incubated with 0.03–10 nM [^3^H]NMS in a final volume of 100 µl buffer (20 mM HEPES, 100 mM NaCl, 10 mM MgCl_2_, pH 7.4) for 4 h at room temperature. For binding interaction assays, cells were incubated with increasing concentrations of ACh in the presence or absence of increasing concentrations of PAMs, and [^3^H]NMS (0.3 nM for the WT, F77^2.56^I, Y179^ECL2^A, W400^7.35^A, and 0.6 nM for Y82^2.61^A M_1_ mAChRs) in a final volume of 100 µl. Atropine at the final concentration of 100 µM was used to determine non-specific binding. The assays were terminated by rapid removal of the radioligand, and two washes with 100 µl/well ice-cold 0.9% NaCl buffer. Radioactivity was determined by addition of 100 µl/well MicroScint scintillation liquid (PerkinElmer Life Sciences, Boston, MA), and counting in a MicroBeta plate reader (PerkinElmer Life Sciences, Boston, MA).

### 2.3 IP-one accumulation assays

The IP-One assay kit (Cisbio) was used for the direct quantitative measurement of myo-Inositol 1 phosphate (IP_1_). Cells were seeded at 25,000 per well into 96-well transparent cell culture plates and incubated overnight at 37 °C. The following day, cells were pre-incubated with IP_1_ stimulation buffer (1 mM CaCl_2_, 0.5 mM MgCl_2_, 4.2 mM KCl, 146 mM NaCl, 5.5 mM D-Glucose, 10 mM HEPES and 50 mM LiCl, pH 7.4) for 1 h before stimulation with ACh in the presence or absence of increasing concentrations of PAMs in IP_1_ stimulation buffer for 1 h at 37 °C, 5% CO_2_. Cells were then lysed with IP_1_ lysis buffer (50 mM HEPES pH 7.0, 15 mM KF, 1.5% V/V Triton-X-100, 3% V/V FBS, 0.2% W/V BSA), and IP_1_ levels were measured by incubation of cell lysates with FRET reagents (the cryptate-labeled anti-IP_1_ antibody and the d2-labeled IP_1_ analogue) for 1 h at 37 °C. The emission signals were measured at 590 and 665 nm after excitation at 340 nm, using the Envision plate reader (PerkinElmer Life Sciences, Boston, MA). Signals were expressed as the FRET ratio: *F =* (fluorescence_665 nm_/fluorescence_590 nm_) × 10^4^, and normalized to the response to maximal ACh concentration (100 µM).

### 2.4 Receptor alkylation assays

Cells were pre-treated with vehicle or the irreversible orthosteric alkylating agent, phenoxybenzamine (PBZ) for 30 min at 37 °C, 5% CO_2_ to reduce functional receptor availability, followed by three extensive washes with PBS. IP_1_ accumulation assays were then performed as described above.

### 2.5 Flow cytometric detection of cell surface receptor expression

Cells were harvested with PBS supplemented with 2 mM EDTA, and transferred to a 96-well v-bottomed plate. Cells were then centrifuged at 350*g* for 3 min at 4 °C and resuspended in 100 µl of blocking buffer (1× HBSS, 5% BSA, 20 mM HEPES, pH 7.4). After 30 min incubation on ice, cells were incubated with mouse monoclonal 9E10 antibody (prepared in-house) targeted to the c-myc epitope tag at 5 µg/ml in assay buffer (1× HBSS, 0.1% BSA, 20 mM HEPES, pH 7.4) for 90 min on ice. Cells were then washed twice with assay buffer and incubated with a secondary goat anti-mouse IgG antibody conjugated to Alexa Fluor 647 (1 µg/ml, Molecular Probes, Invitrogen) for 60 min on ice. Following two washes, cells were resuspended in assay buffer containing 1 µM Sytox blue (Thermo Fisher Scientific). The fluorescence signal was quantified using a FACSCanto II flow cytometer (BD Biosciences).

### 2.6 Data analysis

All data were analysed using GraphPad Prism 7 (San Diego, CA). Inhibition binding data between ACh and the radioligand antagonist, [^3^H]NMS, were analysed according to a one-site binding model [33]. Binding interaction studies between orthosteric agonists and allosteric modulators were fitted to the following allosteric ternary complex model [23] (Eq. (1)):

(1) $$Y=\frac{{\text{B}}_{\max}\left[\text{D}\right]}{\left[\text{D}\right]+\left[\frac{{\text{K}}_{\text{D}}{\text{K}}_{\text{B}}}{\alpha^{\prime }\left[\text{B}\right]+{\text{K}}_{\text{B}}}\right]\left[1+\frac{\left[\text{I}\right]}{{\text{K}}_{\text{I}}}+\frac{\left[\text{B}\right]}{{\text{K}}_{\text{B}}}+\frac{\alpha \left[\text{I}\right]\left[\text{B}\right]}{{\text{K}}_{\text{I}}{\text{K}}_{\text{B}}}\right]}$$

where B_max_ is the total number of receptors, [D], [B] and [I] denote the concentrations of radioligand, allosteric ligand, and orthosteric ligand, respectively, and K_D_, K_B_ and K_I_ represent their respective equilibrium dissociation constants. α′ and α are the cooperativity factors between the allosteric ligand and radioligand or orthosteric ligand, respectively. Values of α or α′ > 1 denote positive cooperativity, values between 0 and 1 denote negative cooperativity, and a value of 1 indicates neutral cooperativity.

To estimate intrinsic efficacy of ACh in IP_1_ accumulation assays at the WT or mutant M_1_ mAChRs, the following operational model of agonism [34] (Eq. (2)) was used:

(2) $$Y=\text{Basal}+\frac{\left({\text{E}}_{\text{m}}-\text{Basal}\right)}{1+\left(\left({\text{K}}_{\text{A}}+\left[\text{A}\right]\right)/\tau \left[\text{A}\right]\right)}$$

where E_m_ is the maximal possible system response and Basal is the response in the absence of agonist. [A] denotes the concentration of ligand, and K_A_ represents its equilibrium dissociation constants. τ denotes the intrinsic efficacy of the ligand, which incorporates the total receptor density and the efficiency of stimulus-response coupling.

Functional interaction studies between orthosteric agonists and allosteric modulators in IP_1_ assays were analysed using the following operational model of allosterism and agonism [35] (Eq. (3)):

(3) $$E=\text{Basal}+\frac{\left({\text{E}}_{\text{m}}-\text{Basal}\right)\left(\left[\text{A}\right]\left({\text{K}}_{\text{B}}+\alpha \beta \left[\text{B}\right]\right)+\tau_{\text{B}}\left[\text{B}\right]\text{E}{\text{C}}_{50}\right)}{\text{E}{\text{C}}_{50}\left({\text{K}}_{\text{B}}+\left[\text{B}\right]\right)+\left(\left[\text{A}\right]\left({\text{K}}_{\text{B}}+\alpha \beta \left[\text{B}\right]\right)+\tau_{\text{B}}\left[\text{B}\right]\text{E}{\text{C}}_{50}\right)}$$

where E_m_ is the maximal possible system response, and Basal is the response in the absence of agonist. K_B_ is the equilibrium dissociation constant of allosteric ligand, and EC_50_ is the concentration of orthosteric agonist required to achieve half maximal response. [A] and [B] denote concentrations of orthosteric and allosteric ligands, respectively. α and β denote allosteric effects on orthosteric ligand binding affinity and efficacy, respectively, and τ_B_ denotes the efficacy of allosteric ligand. This model assumes that ACh is a full agonist at the receptor in both the absence/presence of modulator and/or there is no efficacy modulation (i.e., β = 1). As shown in the Results, both of these assumptions were met depending on the experimental protocol, and thus the β parameter was constrained to 1.

All affinity, efficacy and cooperativity values were estimated as logarithms [36], and where appropriate, were compared using unpaired Student’s *t*-test or one-way analysis of variance (ANOVA) with Dunnett’s multiple comparison test. A p value < 0.05 was considered statistically significant.

## 3 Results

### 3.1 Novel PAMs behave according to a two-state model of allostery at the M_1_ mAChR

**Fig. 2:**
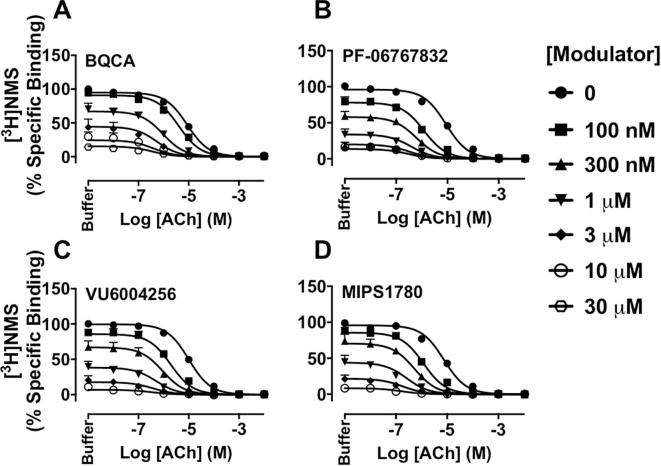
BQCA, PF-06767832, VU6004256 or MIPS1780 display high positive cooperativity with the endogenous agonist ACh and negative cooperativity with the antagonist [^3^H]NMS at the M_1_ mAChR. Inhibition of [^3^H]NMS (0.3 nM) binding by ACh in the presence of increasing concentrations of BQCA (A), PF-06767832 (B), VU6004256 (C) or MIPS1780 (D). Data points represent the mean + S.E.M. of four experiments performed in duplicate. The curves were generated by fitting the data to an allosteric ternary complex model (Eq. (1)). Binding parameter estimates from these experiments are listed in Table 1.

Equilibrium [^3^H]NMS binding interaction studies between ACh and increasing concentrations of BQCA, PF-06767832, VU6004256 or MIPS1780 were performed at the M_1_ mAChR to determine the affinity (K_B_) of each PAM and their binding cooperativity (α) with the endogenous agonist ACh or the radiolabelled antagonist [^3^H]NMS, using an allosteric ternary complex model (Eq. (1)). As shown in Fig. 2, all PAMs displayed high negative cooperativity with the radioligand, but strong positive cooperativity with ACh (Table 1). Consistent with the results of previous studies [21], [22], [26], PF-06767832, VU6004256 and MIPS1780 have higher affinities at the M_1_ mAChR compared to BQCA (Table 1). However, the affinity of BQCA in our study is higher than previously reported values [18], [27], [31], [37] for this compound. This could be due to different experimental conditions including radiolabelled antagonist used, membrane vs. whole cell binding-based assays, incubation time and temperature.

**Table 1:** Binding parameters for the interaction between ACh and M_1_ PAMs, BQCA, PF-06767832, VU6004256 or MIPS1780, at the WT and mutant M_1_ mAChRs. Values represent the mean ± S.E.M. of four experiments performed in duplicate, and were estimated from binding interaction studies using an allosteric ternary complex model (Eq. (1)). The pK_A_ of [^3^H]NMS was constrained to the values listed in Table 3. ND, not determined.

PAMs		pK_B_^a^	Logα (α)_NMS_^b^	Logα (α)_ACh_^c^
BQCA	WT	5.78 ± 0.05	−1.07 ± 0.10 (0.06)	1.32 ± 0.11 (13.5)
	F77^2.56^I	5.82 ± 0.06	−0.90 ± 0.07 (0.08)	1.31 ± 0.12 (13.5)
	Y82^2.61^A	5.76 ± 0.06	−0.84 ± 0.06 (0.11)	1.58 ± 0.11 (30.2)
	Y179^ECL2^A	ND	ND	ND
	W400^7.35^A	ND	ND	ND

PF-06767832	WT	6.51 ± 0.03	−1.05 ± 0.06 (0.06)	1.40 ± 0.08 (16.2)
	F77^2.56^I	6.38 ± 0.07	−1.02 ± 0.12 (0.06)	1.83 ± 0.14* (44.7)
	Y82^2.61^A	5.61 ± 0.12*	−0.16 ± 0.02 (0.62)	1.53 ± 0.11 (30.9)
	Y179^ECL2^A	ND	ND	ND
	W400^7.35^A	ND	ND	ND

VU6004256	WT	6.32 ± 0.03	= −2	1.40 ± 0.09 (15.5)
	F77^2.56^I	6.38 ± 0.03	= −2	1.45 ± 0.10 (14.1)
	Y82^2.61^A	5.65 ± 0.19*	−0.09 ± 0.03 (0.76)	1.08 ± 0.13 (11.2)
	Y179^ECL2^A	ND	ND	ND
	W400^7.35^A	ND	ND	ND

MIPS1780	WT	6.18 ± 0.03	= −2	1.60 ± 0.08 (25.7)
	F77^2.56^I	6.18 ± 0.03	= −2	1.60 ± 0.09 (25.7)
	Y82^2.61^A	5.00 ± 0.24*	−0.16 ± 0.06 (0.69)	1.66 ± 0.19 (45.7)
	Y179^ECL2^A	ND	ND	ND
	W400^7.35^A	ND	ND	ND

a Negative logarithm of the allosteric modulator equilibrium dissociation constant.

b Logarithm of binding cooperativity between [^3^H]NMS and each modulator. Where determined as the preferred model by F-test, logα_NMS_ was constrained to −2, consistent with high negative cooperativity between the two ligands.

c Logarithm of binding cooperativity between ACh and each modulator.

* Significantly different compared to WT, p < 0.05, one-way ANOVA with Dunnett’s post-hoc test.

### 3.2 Novel PAMs modulate ACh affinity at the M_1_ mAChR

**Fig. 3:**
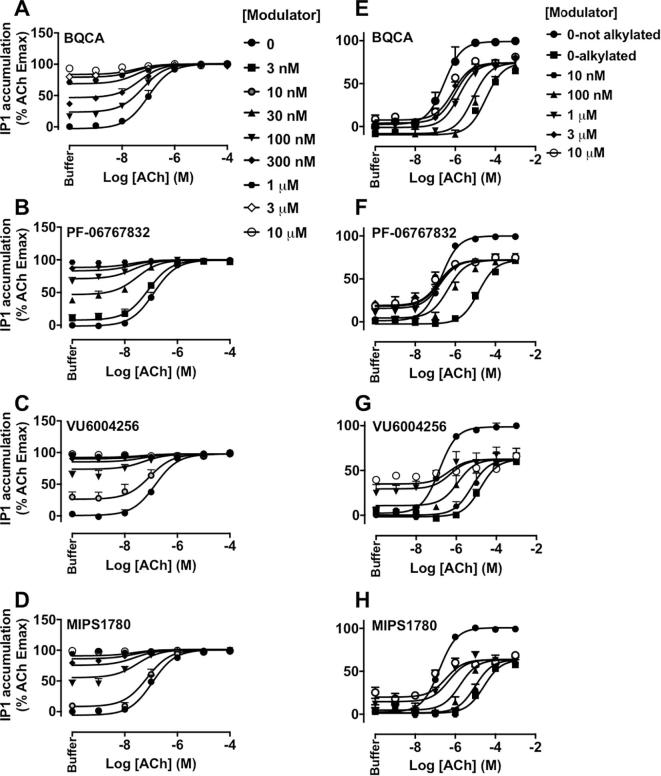
BQCA, PF-06767832, VU6004256 or MIPS1780, modulate the affinity but not efficacy of ACh at the M_1_ mAChR. Modulation of ACh-stimulated IP_1_ accumulation by BQCA (A, E), PF-06767832 (B, F), VU6004256 (C, G) or MIPS1780 (D, H) without PBZ (left panels) or with PBZ (10 µM) pre-treatment (right panels) in CHO-hM_1_ cells. Data points represent the mean + S.E.M. of at least four experiments performed in duplicate. The vehicle-treated curves for ACh under non-alkylation conditions in panels E – H are from a global fit to a three-parameter logistic equation, whereas all other curves in the same panels (under alkylated conditions), as well as the curves in panels A–D, were globally fitted to an operational model of allosterism (Eq. (2)). Functional cooperativity (logαβ) estimates from these experiments are listed in Table 2.

The effects of each modulator on ACh-stimulated IP_1_ accumulation were then investigated. BQCA, PF-06767832, VU6004256 or MIPS1780 robustly enhanced the response to ACh, as indicated by a leftward shift in the ACh concentration response curve, while also stimulating IP_1_ accumulation in their own right (Fig. 3A–D), indicating that they each act as M_1_ mAChR PAM-agonists in our cell line. The functional cooperativity values (αβ) for PF-06767832, VU6004256 or MIPS1780 with ACh were higher than that of BQCA (Table 2). Because ACh was a full agonist in the absence or presence of each PAM, it was unclear whether the allosteric enhancement of ACh potency was due to effects on orthosteric agonist affinity, intrinsic efficacy, or both. Thus, to differentiate these possibilities, IP_1_ interaction studies between ACh and each modulator were also performed under alkylation conditions with PBZ to reduce levels of receptor reserve (Fig. 3E–H). Concentration-response curves to ACh were first generated under pre-treatment of CHO-hM_1_ cells with vehicle or increasing concentrations of PBZ for 30 min (followed by extensive washout) to determine the concentration of PBZ required to reduce receptor availability by approx. 50% for ACh (data not shown). Pre-treatment with 10 µM PBZ reduced the potency and maximal response to ACh in IP_1_ accumulation assays to the level of a partial agonist. This provided a substantial window to unmask any additional effects of the PAMs on ACh maximal response. However, as noted (Fig. 3E–H), BQCA, PF-06767832, VU6004256 or MIPS1780 increased the potency of ACh with no effect on the maximal agonist response, and with αβ values similar to those obtained without PBZ pre-treatment (Table 2). Collectively, these findings indicate that the PAMs exert their allosteric modulatory effects on agonist affinity rather than efficacy (i.e., β = 1).

**Table 2:** Functional cooperativity (logαβ) estimates for the interactions between ACh and M_1_ PAMs in IP_1_ accumulation assays with or without pre-treatment with PBZ in CHO-hM_1_ cells. The values were estimated by fitting the data to an operational model of allosterism (Eq. (3)), and represent the mean ± S.E.M. of at least four experiments performed in duplicate. The pK_B_ of each modulator was constrained to the values listed in Table 1. The β parameter was constrained to 1 to indicate lack of efficacy modulation. Logα β values were not significantly different between non-alkylated and alkylated experiments (p > 0.05, unpaired Student’s *t*-test, degree of freedom 6).

PAM	Logαβ (αβ)	
	Non-alkylated	Alkylated with PBZ
BQCA	1.34 ± 0.10 (22)	1.79 ± 0.11 (62)
PF-06767832	1.92 ± 0.11 (83)	2.16 ± 0.11 (144)
VU6004256	1.78 ± 0.18 (60)	1.96 ± 0.22 (91)
MIPS1780	1.77 ± 0.14 (59)	2.07 ± 0.13 (117)

### 3.3 Effects of amino acid substitutions on receptor expression and affinity of orthosteric ligands at the M_1_ mAChR

The similar mechanism of action observed in aforementioned experiments for the M_1_ PAMs suggest that PF-06767832, VU6004256 and MIPS1780 may bind to a similar binding site as that occupied by BQCA. To further investigate this, and to identify key amino acid residues that govern the binding affinity, efficacy and cooperativity of the novel M_1_ PAMs with ACh, we adopted a structure–function approach. Five key amino acid residues from distinct locations at the M_1_ mAChR, F77^2.56^, Y82^2.61^ in TM2, Y179 in ECL2, Y381^6.51^ in TM6 and W400^7.35^ in TM7 (residues are numbered using the Ballesteros-Weinstein numbering system [38]), previously reported to be involved in the binding of orthosteric, allosteric or bitopic ligands at the M_1_ mAChR, were selected [17], [27], [32], [39], [40].

Whole cell [^3^H]NMS saturation binding assays were first performed to determine the equilibrium dissociation constant of the radiolabelled antagonist (K_D_) and the cell surface receptor expression (B_max_) of the mutant M_1_ mAChRs relative to WT (302549 ± 32197 sites/cell). No [^3^H]NMS binding was observed at Y381^6.51^A (data not shown), therefore, together with the lack of detectable ACh response in functional assays, this mutation was excluded from subsequent experiments. The affinity of [^3^H]NMS was unchanged at F77^2.56^I, Y82^2.61^A, Y179^ECL2^A or W400^7.35^A, however, all the mutations caused a significant reduction in the levels of receptor expression (Table 3). Flow cytometry of antibody binding to the c-myc epitope was also performed to detect immunolabeled cell surface-expressed receptors. The estimated normalized values from saturation binding assays agreed well with the normalized values obtained from flow cytometry experiments (Table 3). The affinity of ACh (K_I_) was estimated from [^3^H]NMS inhibition binding assays using a one-site binding model, and was unaltered at F77^2.56^I or Y179^ECL2^A, mutant receptors, whereas it was significantly reduced at Y82^2.61^A or W400^7.35^A mutant receptors compared with the WT value (Table 3), consistent with our previous observations [31], [32].

**Table 3:** Levels of expression of the mutant M_1_ mAChRs relative to WT in whole cell saturation binding assays and flow cytometric analysis, and equilibrium inhibition binding parameters for [^3^H]NMS and ACh at the WT and mutant M_1_ mAChRs. Values represent the mean ± S.E.M. of four experiments performed in duplicate.

	Receptor expression^a^		pK_A_^b^	pK_I_^c^
	[^3^H]NMS binding	Flow cytometry		
WT			9.63 ± 0.14	5.38 ± 0.03
F77^2.56^I	69.2 ± 7.9*	62.0 ± 5.2*	9.65 ± 0.17	5.41 ± 0.02
Y82^2.61^A	40.0 ± 7.6*	38.9 ± 2.6*	9.28 ± 0.08	5.03 ± 0.05*
Y179^ECL2^A	57.8 ± 11*	57.4 ± 6.0*	9.57 ± 0.18	5.23 ± 0.04
W400^7.35^A	22.1 ± 5.6*	27.0 ± 1.9*	9.80 ± 0.17	4.85 ± 0.05*

a Values represent the percentage of expression relative to the WT receptor expression. Normalised data were not significantly different between estimates of cell surface expression from saturation binding versus flow cytometry of fluorescent antibody labelling of epitope tagged receptors. p > 0.05, unpaired Student’s *t*-test, degree of freedom 6.

b Negative logarithm of the [^3^H]NMS equilibrium dissociation constant.

c Negative logarithm of ACh equilibrium dissociation constant.

* Significantly different compared to WT, p < 0.05, one-way ANOVA with Dunnett’s post-hoc test.

### 3.4 Effects of amino acid substitutions on the binding of PAMs at the M_1_ mAChR

**Fig. 4:**
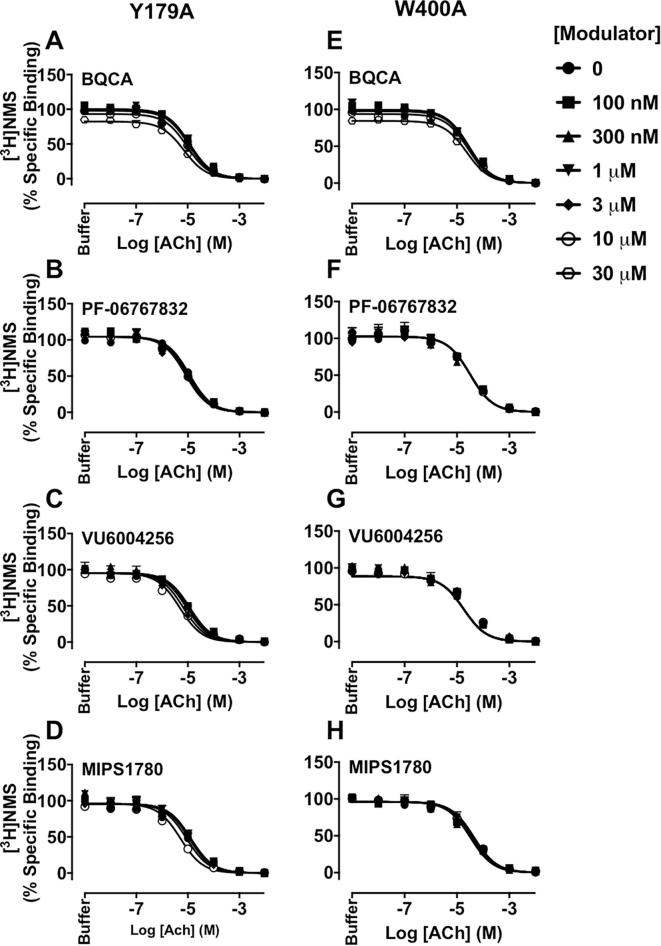
The binding of M_1_ PAMs, BQCA, PF-06767832, VU6004256 or MIPS1780 is abolished at the Y179^ECL2^A or W400^7.35^A M_1_ mAChRs. Inhibition of [^3^H]NMS (0.3 nM) binding by ACh in the presence of increasing concentrations of BQCA (A, E), PF-06767832 (B, F), VU6004256 (C, G) or MIPS1780 (D, H) at the Y179^ECL2^A (left panels) or W400^7.35^A (right panels) mutations. Data points represent the mean + S.E.M. of four experiments performed in duplicate. The curves were generated by fitting the data to an allosteric ternary complex model (Eq. (1)). Binding parameter estimates from these experiments are listed in Table 1.

Equilibrium binding interaction studies were then performed to determine the effects of mutations on the affinity and binding cooperativity of M_1_ PAMs with ACh. At the Y179^ECL2^A or W400^7.35^A mutants, the modulatory effects of BQCA, PF-06767832, VU6004256 or MIPS1780 on ACh inhibition of [^3^H]NMS binding were completely abolished, and hence, their affinity and their binding cooperativity with ACh could not be estimated (Table 1 and Fig. 4), indicating a vital role of these two residues that is common to the actions of all PAM chemotypes tested.

**Fig. 5:**
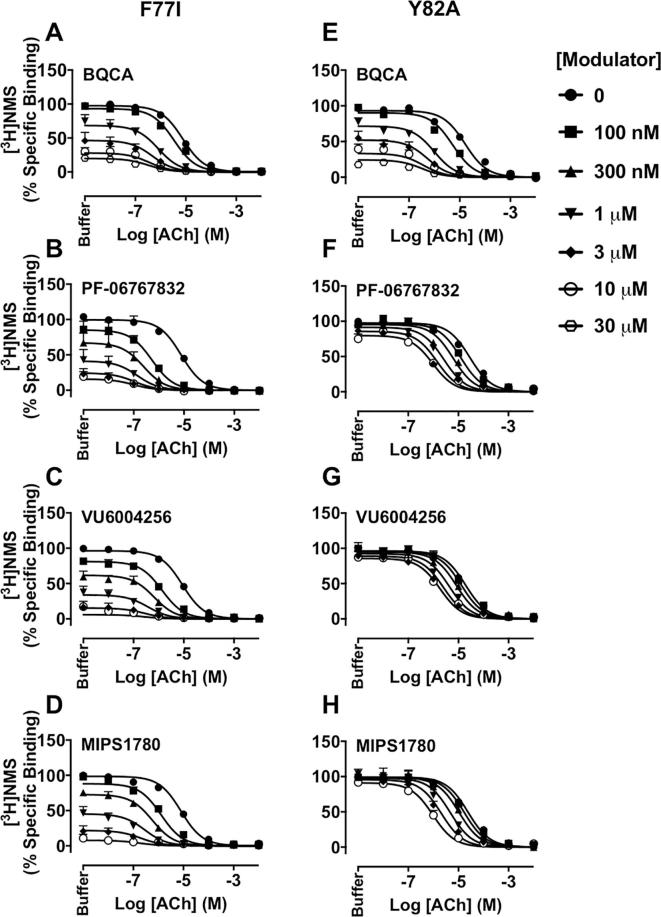
Effects of F77^2.56^I and Y82^2.61^A mutations on the binding affinity and cooperativity of M_1_ PAMs, BQCA, PF-06767832, VU6004256 or MIPS1780, with ACh at the M_1_ mAChR. Inhibition of [^3^H]NMS binding by ACh in the presence of increasing concentrations of BQCA (A, E), PF-06767832 (B, F), VU6004256 (C, G) or MIPS1780 (D, H) at the F77^2.56^I (left panels) or Y82^2.61^A (right panels) mutations. Assays were performed using K_A_ concentrations of [^3^H]NMS (0.3 nM and 0.6 nM for F77^2.56^I and Y82^2.61^A, respectively). Data points represent the mean + S.E.M. of four experiments performed in duplicate. The curves were generated by fitting the data to an allosteric ternary complex model (Eq. (1)). Binding parameter estimates from these experiments are listed in Table 1.

F77^2.56^ was previously reported to be important for the binding and agonist activity of bitopic ligands i.e., extended hybrid molecules that concomitantly engage both orthosteric and allosteric sites [32], [39], [40]. As shown in Table 1 and Fig. 5A–D, the F77^2.56^I mutation did not alter the affinity of the PAMs when compared with WT. The binding cooperativity between ACh and the PAMs was also unchanged, with the exception of PF-06767832, which displayed a significant, albeit small, increase in cooperativity with ACh at this mutation (Table 1). Collectively, these data suggest that a bitopic mechanism of action is unlikely for these compounds although the residue at position 2.56 may play some role in the transmission of cooperativity depending on the nature of the interacting ligands. Interestingly, at Y82^2.61^A, the affinities of PF-06767832, VU6004256 or MIPS1780, but not BQCA, were significantly reduced (Fig. 5E–H), suggesting that the PAMs may be adopting slightly different binding poses within a shared pocket. Furthermore, while the binding cooperativity with [^3^H]NMS of PF-06767832, VU6004256 or MIPS1780, but not BQCA, was abolished at Y82^2.61^A, the binding cooperativity values between ACh and the PAMs were not different from the WT values (Table 1).

### 3.5 Effects of amino acid substitutions on signaling properties of PAMs at the M_1_ mAChR

We next determined the effects of selected mutations on the potency (pEC_50_) and efficacy (τ_A_) of ACh at each mutant receptor in parallel to the WT M_1_ mAChR in IP_1_ accumulation assays. To account for the effect of varying receptor expression levels of the different constructs on the efficacy of ACh, the estimated τ_A_ values were corrected for receptor expression relative to the WT M_1_ mAChR B_max_ value. As shown in Table 4, while all mutations reduced ACh potency, when corrected for expression relative to WT, the estimated operational efficacy of the cognate agonist was significantly increased at the Y82^2.61^A and the W400^7.35^A mutants, indicating an important role of these residues in the transmission of signaling efficacy. In contrast, efficacy was unchanged at the F77^2.56^I or Y179^ECL2^A mutant receptors.

**Table 4:** Potency (pEC_50_) and efficacy (τ_A_) of ACh in IP_1_ accumulation assays at the WT and mutant M_1_ mAChRs. Values represent the mean ± S.E.M. of at least four experiments performed in duplicate. Potency values were estimated using a three-parameter logistic equation, and efficacy values were quantified according to an operational model of agonism (Eq. (2)). The pK_A_ of ACh at each mutant M_1_ mAChR was constrained to the corresponding binding affinity (pK_I_), listed in Table 3.

	pEC_50_^a^	Logτ_A_ (τ_A_)^b^
WT	6.82 ± 0.03	1.47 ± 0.02 (30)
F77^2.56^I	6.52 ± 0.06*	1.67 ± 0.03 (47)
Y82^2.61^A	5.72 ± 0.04*	2.39 ± 0.08* (245)
Y179^ECL2^A	5.90 ± 0.03*	1.42 ± 0.06 (26)
W400^7.35^A	5.43 ± 0.04*	3.78 ± 0.17* (6025)

a Negative logarithm of the agonist concentration required to produce half the maximal response, estimated using a three-parameter logistic equation.

b Logarithm of the functional efficacy of ACh, estimated via fitting to an operational model of agonism (Eq. (1)) and then corrected for receptor expression levels relative to WT.

* Significantly different compared to WT, p < 0.05, one-way ANOVA with Dunnett’s post-hoc test.

**Fig. 6:**
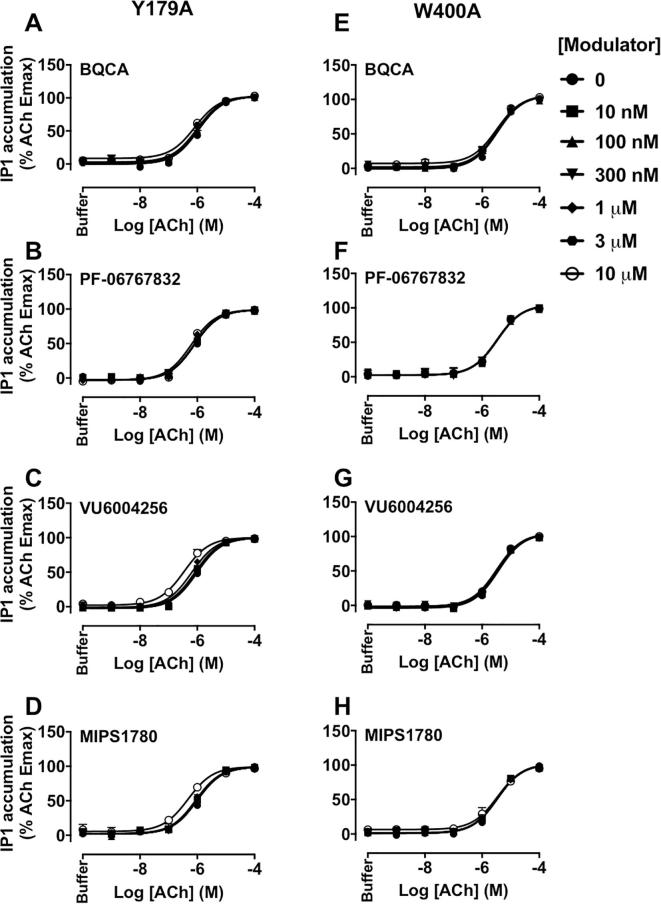
The modulation of ACh-stimulated IP_1_ accumulation by M_1_ PAMs, BQCA, PF-06767832, VU6004256 or MIPS1780 is abolished at the Y179^ECL2^A or W400^7.35^A M_1_ mAChRs. IP_1_ interaction assays between ACh and increasing concentrations of BQCA (A, E), PF-06767832 (B, F), VU6004256 (C, G) or MIPS1780 (D, H) at the Y179^ECL2^A (left panels) or W400^7.35^A (right panels) mutations. Data points represent the mean + S.E.M. of four experiments performed in duplicate. The curves were generated by fitting the data to an operational model of allosterism (Eq. (3)). Operational parameter estimates from these experiments are listed in Table 5.

**Fig. 7:**
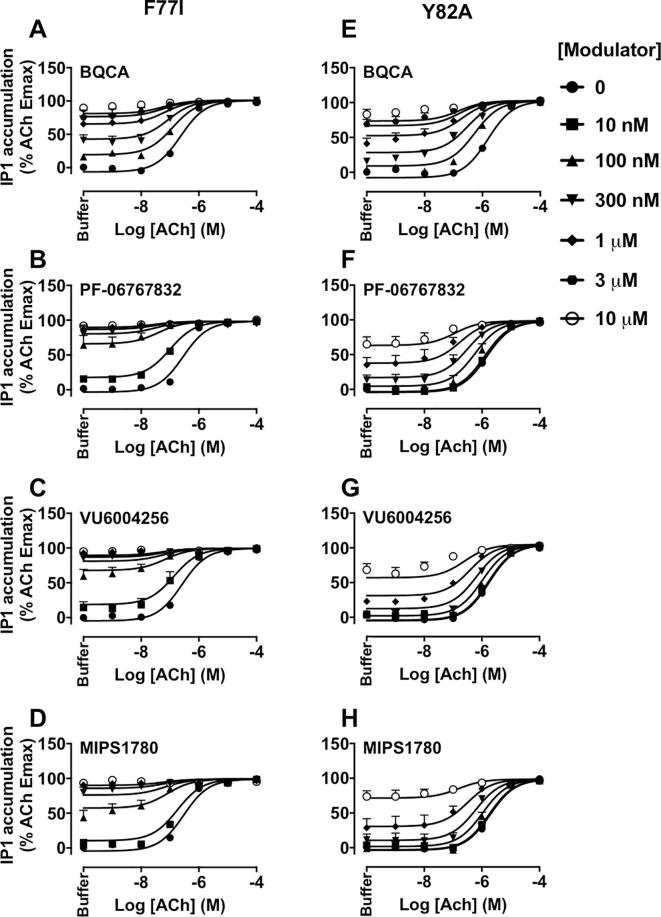
Effects of F77^2.56^I and Y82^2.61^A mutations on the efficacy and functional cooperativity of M_1_ PAMs, BQCA, PF-06767832, VU6004256 or MIPS1780, with ACh at the M_1_ mAChR. IP_1_ interaction assays between ACh and increasing concentrations of BQCA (A, E), PF-06767832 (B, F), VU6004256 (C, G) or MIPS1780 (D, H) at the F77^2.56^I (left panels) or Y82^2.61^A (right panels) mutations. Data points represent the mean + S.E.M. of four experiments performed in duplicate. The curves were generated by fitting the data to an operational model of allosterism (Eq. (3)). Operational parameter estimates from these experiments are listed in Table 5.

Finally, the effects of mutations on the efficacy (τ_B_) of the PAMs and their functional cooperativity with ACh were investigated in IP_1_ interaction experiments. The estimated τ_B_ values were corrected for receptor expression of mutant receptors relative to the WT M_1_ mAChR B_max_ value. At the WT M_1_ mAChR, BQCA, PF-06767832, VU6004256 or MIPS1780 behaved as PAM-agonists (Table 5 and Fig. 3A–D). The Y179^ECL2^A or W400^7.35^A mutations resulted in a loss of ACh potentiation by all PAMs tested (Fig. 6), in agreement with the binding experiments, again highlighting a common and critical role for these residues irrespective of the PAM scaffold. The functional efficacy of BQCA, PF-06767832 or MIPS1780 was increased, whereas the efficacy of VU6004256 was unchanged at the F77^2.56^I mutation. However, their cooperativities with ACh were not different at this construct compared to the WT M_1_ mAChR (Table 5 and Fig. 7A–D). As with the interpretation of the binding interaction studies (Table 1), the modest effects of this mutation argue against a bitopic mechanism of action for the novel chemotypes. At the Y82^2.61^A construct, the functional cooperativity values of PAMs with ACh were similar to the WT values. Interestingly, this mutation had differential effects on the efficacy of the PAMs, causing a significant decrease in VU6004256 efficacy while increasing the efficacy of BQCA or MIPS1780, and not altering PF-06767832 efficacy relative to WT (Table 5 and Fig. 7E–H).

**Table 5:** Operational model parameters for IP_1_ interaction assays between ACh and M_1_ PAMs, BQCA, PF-06767832, VU6004256 or MIPS1780, at the WT and mutant M_1_ mAChRs. Values represent the mean ± S.E.M. of at least four experiments performed in duplicate, and were estimated using an operational model of allosterism (Eq. (2)). The pK_B_ of each modulator was constrained to the corresponding binding affinity, listed in Table 1. The β parameter was constrained to 1 to indicate lack of efficacy modulation. ND, not determined (no ACh modulation).

PAMs		Logαβ (αβ)^a^	Logτ_B_ (τ_B_)^b^
BQCA	WT	1.34 ± 0.10 (22)	0.78 ± 0.03 (6)
	F77^2.56^I	1.40 ± 0.15 (25)	1.03 ± 0.06* (11)
	Y82^2.61^A	1.66 ± 0.11 (46)	1.30 ± 0.07* (20)
	Y179^ECL2^A	ND	ND
	W400^7.35^A	ND	ND

PF-06767832	WT	1.92 ± 0.11 (83)	1.01 ± 0.03 (10)
	F77^2.56^I	2.05 ± 0.13 (112)	1.51 ± 0.07* (32)
	Y82^2.61^A	1.66 ± 0.12 (46)	0.95 ± 0.12 (9)
	Y179^ECL2^A	ND	ND
	W400^7.35^A	ND	ND

VU6004256	WT	1.78 ± 0.18 (60)	1.24 ± 0.05 (17)
	F77^2.56^I	1.71 ± 0.15 (51)	1.43 ± 0.06 (27)
	Y82^2.61^A	1.29 ± 0.10 (19)	0.50 ± 0.10* (3)
	Y179^ECL2^A	ND	ND
	W400^7.35^A	ND	ND

MIPS1780	WT	1.77 ± 0.14 (59)	1.00 ± 0.05 (10)
	F77^2.56^I	1.80 ± 0.15 (63)	1.52 ± 0.06* (33)
	Y82^2.61^A	1.96 ± 0.14 (91)	1.85 ± 0.12* (71)
	Y179^ECL2^A	ND	ND
	W400^7.35^A	ND	ND

a Logarithm of functional cooperativity between ACh and each modulator.

b Logarithm of functional efficacy of the modulator corrected for receptor expression levels relative to WT.

* Significantly different compared to WT, p < 0.05, one-way ANOVA with Dunnett’s post-hoc test.

## 4 Discussion

Subtype selective allosteric modulation of M_1_ mAChRs has been increasingly explored as a potential approach for the treatment of AD and other cognitive deficit disorders [7], [41]. The discovery of BQCA, a highly selective M_1_ mAChR PAM [17] led to development of newer classes of M_1_ PAMs with different pharmacological and pharmacokinetic properties, including PF-06767832 [21], VU6004256 [24] and MIPS1780 [26]. Despite the distinct chemical scaffolds of these PAMs, the current study provides strong support for a common mode of binding to that seen with BQCA, and a similar mode of action whereby cooperativity arises predominantly from modulation of ACh affinity. This has implications for the discovery of novel M_1_ mAChR-targeting PAMs with improved drug-like properties to facilitate translational studies.

There is a substantial body of pharmacology data that supports the existence of at least two allosteric sites on the mAChRs. By far the best studied has been the so-called “common” allosteric binding site, located in an extracellular vestibule above the orthosteric pocket and predominantly comprised of residues in ECL2, TM2 and TM7 [42]. This region is recognized by prototypical and well-studied negative allosteric modulators (NAMs), such as gallamine and C_7_/3-phth [43], but also by PAMs such as BQCA (M_1_) [27] and LY2033298 (M_2_ and M_4_; [23], [44], [45]). The existence of this vestibular site has been more recently validated directly through structural and computational studies of the M_1_–M_4_ mAChRs [42], [46], [47], [48], [49]. In contrast, the location of the “second” allosteric site that binds indolocarbazole and benzimidazole modulators is currently unknown, although one study has suggested a potential intracellular pocket [28], [50], [51].

To elaborate our understanding of novel M_1_ mAChR PAMs, we adopted two complementary approaches to characterize the nature of the allosteric action and likely binding mode of these compounds. The first approach characterised affinity, relative efficacy and cooperativity with orthosteric ligands using a combination of cell-based assays of M_1_ mAChR-mediated IP_1_ accumulation and radioligand binding. Moreover, we extended this analysis to account for the contribution of receptor reserve in the observed pharmacology. We have previously shown that the irreversible receptor alkylating agent, PBZ, precludes the binding of ACh directly, or indirectly prevents the binding of PAMs to the allosteric site via high negative cooperativity [52]. In either instance, the net effect is a reduction in receptor reserve such that the functional affinity and relative efficacy of each agent as a direct activator of the M_1_ mAChR can be determined. Moreover, interaction studies between ACh and each PAM under these same conditions, where ACh would be acting as essentially a partial agonist, provided a powerful functional approach to directly determine whether the PAMs exert effects on agonist affinity, intrinsic efficacy, or both as part of their allosteric mechanism of action. Accordingly, three key outcomes were obtained from the functional and binding experiments. First, each of the PAMs has the potential to display intrinsic allosteric agonism (i.e., as PAM-agonists), second, allosteric effects were mediated primarily through the enhancement of agonist binding affinity and, third, all modulators exhibited high positive cooperativity with ACh but high negative cooperativity with the antagonist, [^3^H]NMS. These observations mirror those previously noted with BQCA and the related BQZ-12 (3-((1*S*,2*S*)-2-hydroxycyclohexyl)-6-((6-(1-methyl-1*H*-pyrazol-4-yl)pyridin-3-yl)methyl)benzo[*h*]quinazolin-4(3*H*)-one)), [18], [31], [52], [53]. Collectively, our findings suggest that PAMs acting via the “common” allosteric pocket are likely to mimic the two-state mode of allosteric effect described for BQCA [19], [54]. The work also supports the importance of understanding relative intrinsic agonism of modulators, as the observed degree of PAM-agonism can be highly system dependent [54], and this property could contribute to undesirable over-activation of a target GPCR by novel classes of PAMs.

In the second series of studies, we probed for binding site location via mutational analysis of key residues previously implicated in the actions of prototypical “common” site modulators or bitopic ligands. Our previous mutagenesis and molecular modelling predicted important roles for Y179^ECL2^ and W400^7.35^ in the stability of BQCA binding via hydrophobic π-π interactions [27]. We had also confirmed the importance of these residues for BQZ-12 binding, indicating a shared binding site for both ligands [31]. In the current study, we also found that Y179^ECL2^ and W400^7.35^ are absolutely critical for the binding and activity of PF-06767832, VU6004256 and MIPS1780, consistent with the binding of these PAMs to a similar site as that of BQCA or BQZ-12. Further support for these residues, as well as for Y82^2.61^, was noted in a prior homology model of the M_1_ mAChR bound to a structurally related compound to PF-06767832 (compound **11** in [21]). We had also previously found that Y82^2.61^ was involved in transmission of positive cooperativity and/or modulator binding affinity in studies focusing on BQCA and BQZ-12 [27], [31]. Our current findings with PF-06767832, VU6004256 and MIPS1780 are also in agreement with a broad role for this residue either in modulator binding affinity and/or transmission of cooperativity. The reduced binding cooperativity of these modulators with [^3^H]NMS but not with ACh at Y82^2.61^A indicates the key role of this residue in the transmission of cooperativity specifically with the antagonist and not the agonist. The differences in which pharmacological behaviours are modified (i.e., affinity, cooperativity or efficacy), likely relate to differences in the poses each PAM adopts within the common allosteric pocket. Our data suggest that Y82^2.61^ may form key π-π interactions with the thiazole (in PF-06767832) or pyrazole (in VU6004256 and MIPS1780) pendent group, whereas Y179^ECL2^ and W400^7.35^ interact with the core of these compounds as well as BQCA, which lacks an extended pendent group. However, this needs to be confirmed in future studies by additional structure-activity relationships and molecular modelling. In contrast to the aforementioned key residues, only modest effects of the F77^2.56^I mutation on the M_1_ PAMs were noted. Previous studies had highlighted a vital role for this TM2 residue in the action of extended, bitopic, ligands [32], [39], [40]. The lack of observed effect in the current study argues against such a mode of interaction for the novel chemotypes investigated herein. Ideally, an additional test of our hypothesis for a common binding site for different PAM chemotypes at the M_1_ mAChR would be a direct competition assay between each PAM and a neutral, or near neutral, allosteric ligand binding to the same site with high affinity. However, to our knowledge, no such allosteric ligand has thus far been identified for the M_1_ mAChR.

Collectively, our findings support a model whereby PF-06767832, VU6004256 and MIPS1780 share a similar binding site with BQCA, located within the “common” extracellular vestibule region. However, additional studies are required to better understand the structural basis of selectivity of these PAM scaffolds. For instance, W^7.35^ is conserved across the five mAChR subtypes, Y179^ECL2^ is present in both M_1_ and M_2_ mAChRs, and Y^2.61^ is conserved across all but the M_3_ subtype indicating complexity beyond sequence conservation in the subtype selective actions of the modulators. One intriguing possibility is that the exquisite degrees of selectivity achieved by allosteric modulators of GPCRs arise from a combination of both allosteric pocket residues *and* energetically preferred dynamic networks that underlie transmission of cooperativity between orthosteric and allosteric sites [20], [46], [55], [56]. In addition, despite the commonalities in mechanism proposed for the different classes of M_1_ PAMs described in our study, it should be noted that different modes of GPCR allosteric modulation occur, including differential effects on efficacy in addition to affinity, as well as the potential for pathway-biased modulation [23], [45], [52], [57], highlighting additional mechanistic questions for the field. Our current study was limited to analysis of the relative effects on a single signaling endpoint and additional work is required to understand the extent to which biased modulation may or may not occur. Nonetheless, and as evidenced by very recent advances in structure-based approaches to discovering new allosteric modulators [42], [58], [59], novel insights into the structural basis of M_1_ allosteric modulator binding and activity can facilitate the rational design of new PAMs as drug-like candidates.

## 5 Conflict of interest

The authors declare no conflict of interest.
